# Identification and analysis of mitochondria-related key genes of heart failure

**DOI:** 10.1186/s12967-022-03605-2

**Published:** 2022-09-07

**Authors:** Haozhen Yu, Mujun Yu, Zhuang Li, Enhu Zhang, Heng Ma

**Affiliations:** 1 School of Basic Medical Sciences, Shaanxi University of Chinese Medicine, Xianyang, Shaanxi China; 2 School of Life Sciences, Yan’an University, Yan’an, China; 3 Department of Physiology and Pathophysiology, School of Basic Medicine, Fourth Military Medical University, Xi’an, China

**Keywords:** Heart failure, Mitochondria, Competing endogenous RNA (ceRNA) network, Key genes, Bioinformatics

## Abstract

Mitochondria-induced cell death is a vital mechanism of heart failure (HF). Thus, identification of mitochondria-related genes (Mito-RGs) based on transcriptome sequencing data of HF might provide novel diagnostic markers and therapeutic targets for HF. First, bioinformatics analysis was conducted on the GSE57338, GSE76701, GSE136547, and GSE77399 datasets in the Gene Expression Omnibus. Next, we analyzed HF-Mito differentially expressed genes (DEGs) using the protein–protein interaction (PPI) network for obtaining critical genes and exploring their functions. Subsequently, immune cell scores of the HF and normal groups were compared. The potential alteration mechanisms of the key genes were investigated by constructing a competing endogenous RNA network. Finally, we predicted potential therapeutic agents and validated the expression levels of the key genes. Twenty-three HF-Mito DEGs were acquired in the GSE57338 dataset, and the PPI network obtained four key genes, including *IFIT3*, *XAF1*, *RSAD2*, and *MX1*. According to gene set enrichment analysis, the key genes showed high enrichment in myogenesis and hypoxia. Immune cell analysis demonstrated that aDCs, B cells, and 20 other immune cell types varied between the HF and normal groups. Moreover, we observed that H19 might affect the expression of *IFIT3*, *AXF1*, and *RSAD2*. PCGEM1 might regulate *RSAD2* expression. A total of 515 potential therapeutic drugs targeting the key genes, such as tretinoin, silicon dioxide, and bisphenol A, were acquired. Finally, *IFIT3*, *RSAD2*, and *MX1* expression increased in HF samples compared with normal samples in the GSE76701 dataset, conforming to the GSE57338 dataset analysis. This work screened four key genes, namely, *IFIT3, XAF1, RSAD2*, and *MX1*, which can be further explored in subsequent studies for their specific molecular mechanisms in HF.

**Supplementary Information:**

The online version contains supplementary material available at 10.1186/s12967-022-03605-2.

## Background

Heart failure (HF) is a clinical disorder characterized by insufficient tissue perfusion and congestion of systemic or pulmonary circulation, caused by impaired systolic or diastolic cardiac function and decreased ejection and filling ability. Myocardial ischemia and infarction developing into HF are still the leading causes of its high incidence rate and mortality despite tremendous progress in cardiovascular medicine and medical procedures. The prevalence of HF is 1–2% worldwide, and patients over 70 years account for more than 10% [1] of the total HF patients. The prognosis of HF is poor, and the mortality rate within 4–5 years is approximately 50% [2], causing a heavy social and economic burden. The estimated cost of HF as a share of total health care expenditure is expected to increase every year in developed economies. Many interrelated conditions eventually lead to HF, and these include diabetes, obesity, and hypertension. HF involves alterations in energy metabolism and electroconductibility; as a result, the heart cannot meet circulatory requirements [3, 4]. The widely recognized variant of HF is “systolic function–preserved” or “diastolic” HF, which has the feature of ventricular filling resistance but not systolic defects. Obesity, ischemia, aging, hypertension, and diabetes are the most common causes of HF with preserved ejection fraction [5]. The incidence of HF may increase as time goes by in terms of the future. However, the underlying mechanism remains unclear.

Mitochondria are double membrane–enclosed organelles present in almost all eukaryotes. Mitochondria primarily produce adenosine triphosphate (ATP) through the process of oxidative phosphorylation. Mitochondrial membrane potential (MMP) and intimal impermeability are the key characteristics of functional mitochondria. For maintaining oxidative phosphorylation, the metabolism of various carbon substrates is needed through certain pathways, finally converging with the tricarboxylic acid (TCA) cycle for producing reduction equivalent. Mitochondria contain an active calcium transport system, and many enzymes related to the oxidative metabolic pathway can be activated by calcium. Mitochondria support life and are related to cell death initiation. Mitochondria-induced cell death is an essential mechanism of HF [6]. The relationship of mitochondrial calcium level with cardiac insufficiency in the process of chronic HF has become a research hotspot. Mitochondrial function has a critical effect on HF pathophysiology.

In the present study, mitochondria-related genes were identified in HF patients using transcriptome sequencing–based bioinformatics analysis to provide novel ideas for diagnosing and treating the disease.

## Methods

### Data source

The HF-related expression data were acquired from the GSE57338, GSE76701, GSE136547, and GSE77399 datasets, of which the GSE57338 dataset had 177 HF cases and 136 normal samples; GSE76701 dataset had eight samples (HF: Normal = 4:4); GSE136547 dataset had 80 samples (HF: Normal = 48:32); and HF: Normal ratio was 13:12 in the GSE77399 dataset. A total of 1576 mitochondria-related genes (Mito-RGs) were obtained from the molecular signatures database (MSigDB) (http://software.broadinstitute.org/gsea/msigdb) [6].

### DEGs detection in normal versus HF samples

We performed differential analysis to screen DEGs between HF samples (n = 177) and normal samples (n = 136) from the GSE57338 dataset with “limma” R package [7], with *P* < 0.05 and |log2(fold change, FC)| > 0.5 as thresholds [8]. We used R package “ggplot2” (version 3.3.5) [9] and “pheatmap” (version 1.0.12) to draw volcano plots and heat maps, respectively, to show DEGs expression.

### Identification of HF-related genes (HFGs) by weighted gene co-expression network analysis (WGCNA)

WGCNA has been extensively utilized in trait and gene association analysis [10]. In this work, we utilized “WGCNA” R package [10] for constructing a co-expression network by considering the gene expression levels of the 313 samples in GSE57338 as input data and HF and normal as trait data. First, samples clustering was performed by using hclust function to recognize outliers, and the parameter was set to “method = average” to calculate the distance. Moreover, the optimal soft threshold was approximate scale-free network. Modules were segmented using a dynamic shear tree algorithm, and the modules associated with HF were identified using correlation analysis. HFGs were obtained through Module Membership (MM) and Gene Significance (GS) within the modules [10].

### Identification of HF-mitochondria-related DEGs (HF-Mito DEGs)

The HF-Mito DEGs were obtained by intersecting the Mito-RGs, HFGs, and DEGs detected from the GSE57338 dataset, and the expression of HF-Mito DEGs in HF as well as normal group was analyzed by the Wilcox test method. Then, the R package “clusterProfiler” [11] was utilized for GO and KEGG analyses of HF-Mito DEGs, and the top five GO and top 10 KEGG signaling pathways were displayed.

### Identification of key genes and gene set enrichment analysis (GSEA)

We used STRING (https://string-db.org) website to construct a protein–protein interaction (PPI) network of HF-Mito DEGs with a confidence level of 0.4 (medium confidence = 0.4), and the relationship pairs were obtained after removing discrete proteins, imported into Cytoscape software for visualization. Then, molecular complex detection (MCODE) was used for analysis, and the degree cutoff was set to 2, node score to 2, k-score to 2, and Max. Depth to 100 to classify the gene network clusters and obtain key genes. Afterward, 177 HF patients were classified into low- or high-expression group based on the median key gene levels, and GSEA analysis was performed for all genes with set thresholds of SIZE > 20 and NOM. *P* < 0.05.

### Immune cells scores analysis in HF and normal groups

Using the 29 immune-related gene sets, the immune activity of each sample can be obtained accurately. First, this work utilized “GSVA” R package [12] for calculating immune gene set contents in 177 HF samples and 136 normal samples and analyzed the differences in immune gene sets between the HF and normal samples. In addition, correlations between the key genes and the differential immunity genes were analyzed.

### Drug prediction analysis

The comparative toxicogenomics database (CTD) helps understand the complex network of interactions between genes and proteins. The current study used the CTD database to predict potential therapeutic agents for the key genes.

### Competing endogenous RNA network establishment based on the key genes

Differentially expressed miRNAs and lncRNAs were analyzed in normal and HF samples from the GSE136547 dataset and the GSE77399 dataset by R package “limma” [7], upon screening criteria of *P* < 0.05 and |log2(FC)| > 0.5. Then, miRWalk (http://mirwalk.umm.uni-heidelberg.de/) was utilized for predicting the key genes to obtain the miRNAs that bind to their targets, setting the parameters as follows: Score = 0.95; Position = 3UTR; and miRDB = 1. These miRNAs were intersected with the differential miRNAs to obtain the common miRNAs. Next, the starBase database was used to predict lncRNAs interacting with the common miRNAs, and the predicted lncRNAs were intersected with the differential lncRNAs to obtain the common lncRNAs. Finally, the miRNAs and the key genes with regulatory relationships were extracted based on the final lncRNAs, and the network of the key genes, miRNAs, and lncRNAs with regulatory relationships was obtained using Cytoscape software.

### Validation of critical gene levels

The present study confirmed critical gene expression from the GSE76701 dataset using Wilcoxon test method. The expression box line plots of the key genes in HF and normal groups were plotted using the “ggplot” R package.

### Animals

All animal experiments using 6–8-week-old wild-type (WT) male C57BL/6 MICE gained approval from the Animal Ethical Laboratory Committee of the Fourth Military Medical University. The mice were placed in a temperature-controlled chamber (22 ± 2 °C) for 12 h of light/dark cycle and were free to obtain food and water.

### Heart failure model

A murine heart failure model was established as previously described. We used 6–8-week-old WT male C57BL/6 mice. After adequate anesthesia attained by intraperitoneal injection of 1% sodium pentobarbital (50 mg/kg), the mice were placed in the supine position on a fixed plate. The upper thorax region was shaved and the tongue was retracted. Then, a 24-gauge i.v. catheter was inserted into the trachea. The catheter was subsequently connected to a small animal ventilator (HX-101E, Techman Soft Co., Ltd., Chengdu, China) via the Y-shaped connector. The mice were ventilated with a tidal volume of 2.4 mL, and their respiratory rate was 120 breaths per minute. To properly expose the heart, a left thoracotomy was performed by separating the third and the fourth intercostal spaces. After connecting the electrocardiogram, left thoracotomy was performed to expose the heart for the ligation of the left anterior descending coronary artery 2 to 3 mm after the origin of the coronary artery. Standard II lead electrocardiogram (ECG) was observed; ST segment and/or T wave elevation or decrease, local color darkening of the heart, and other myocardial ischemia changes were observed as signs of successful ligation, and the chest was closed. The mice used in the experiment were self-adaptive raised for four weeks under normal diet since the purchase. Echocardiography was performed before model establishment and at 4 weeks. Four chamber sections of the left ventricular long axis, short axis, and apex were routinely obtained, and the anterior wall, left indoor diameter, and posterior wall of the left ventricle were observed, and ejection fraction was calculated. Ejection fraction below 50% was considered HF. Specific echocardiographic results are presented in supplementary materials.

### Validation of the expression levels of critical genes by RT-qPCR

cDNA from the 4-week time point (sham, HF n = 12 C57BL/6 mice left ventricle) generated as described above was used for qPCR. Primers spanning exon–exon junctions were designed using Primer-BLAST (https://www.ncbi.nlm.nih.gov/tools/primer-blast/). GAPDH was used as the reference (housekeeping) gene. The qPCR assays were performed using FastStart Universal SYBR Green Master mix (Takara Biomedical Technology (Beijing) Co.) in line with the manufacturer’s protocol with minor modifications. A 25-µL reaction (10 ng cDNA) in the 96-well plate (Axygen Scientific Inc, Silicon Valley) was performed on a CFX96TMReal-Time System (BIO-RAD). Primer sequences are shown in Table 1.

**Table 1:** Primer sequences

Primer name	Sequence (5′–3′)
*Ifit3*(Mus)-F	TGAACTGCTCAGCCCACAC
*Ifit3*(Mus)-R	AATGGCACTTCAGCTGTGGA
*Xaf1*(Mus)-F	TCCACTTCATGCTCCACGAG
*Xaf1*(Mus)-R	GTTGGCTTTCCTTGGTCTGC
*Rsad2*(Mus)-F	CCTGTGCGCTGGAAGGTTT
*Rsad2*(Mus)-R	TTCAGGCACCAAACAGGACA
*Mx1*(Mus)-F	CCTCCCACATCTGTAAATCACTG
*Mx1*(Mus)-R	CGGTTTCCTGTGCTTGTATCA

### Statistical analysis

Bioinformatics statistical analysis was performed in R language (version 3.6.3). Quantitative results were examined through Prism 9.0, and represented by mean ± SEM. Student’s two-tailed, unpaired *t* test was adopted to analyze differences between the groups, with *P* < 0.05 indicating statistical significance.

## Results

### Identification of differentially expressed genes (DEGs) between HF and normal samples

**Fig. 1:**
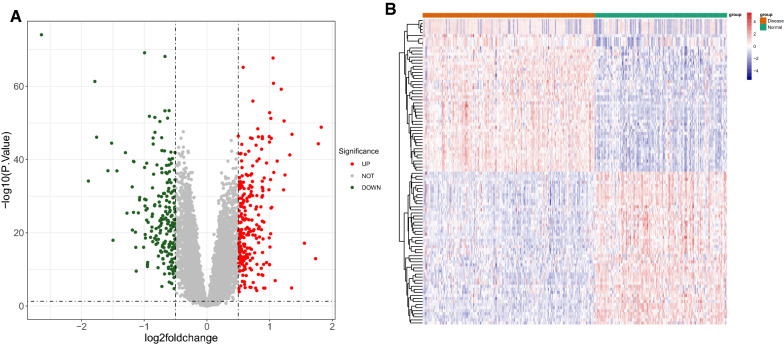
Identification of DEGs between normal and HF groups. **A** Volcano plot showing DEGs in HF vs. the normal group; **B** Heat map of Top 50 increased and decreased DEGs (green indicates normal sample and red indicates HF sample)

A total of 450 DEGs between HF and normal samples were identified in the GSE57338 dataset with the cutoff value of *P* < 0.05 and |log2(fold change, FC)| > 0.5, including 244 genes showing upregulation and 206 genes showing downregulation (Additional file 1: Table S1 and Additional file 2: Table S2). Volcano plots and heat map results of the DEGs are shown in Fig. 1A, B.

### Identification of HF-related genes (HFGs) by weighted gene co-expression network analysis (WGCNA)

**Fig. 2:**
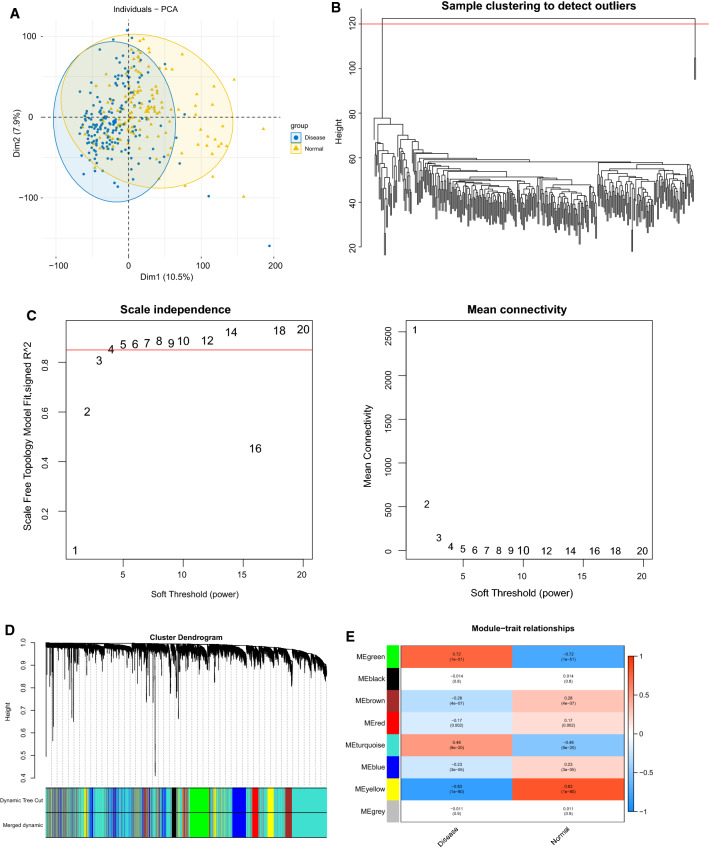
WGCNA performed for identifying gene modules significantly related to HF. **A** The PCA results of samples clustering; **B** sample clustering; **C** Heat map of sample clustering and its characteristics; **D** Filtering of soft thresholds; **E** Division of gene modules

First, the height cutoff value was set at 120, and all samples were clustered based on their Euclidean distance to detect good samples and genes. The PCA results are shown in Fig. 2A. Two outlier samples, GSM1380018 and GSM1379815, were eliminated, and the remaining 311 samples were used for subsequent analysis (Fig. 2B). The optimal soft threshold value of five was determined from Fig. 2C, and the dynamic shear tree algorithm was utilized to segment the modules at the min module size of 300 to obtain eight modules (Fig. 2D). The genes in each module are shown in Additional file 3: Table S3. Given that the correlation analysis demonstrated that three modules—yellow, green, and turquoise—strongly correlated with HF, the genes in these three modules were regarded as HF-related genes (Fig. 2E).

### Identification of HF-Mito DEGs

**Fig. 3:**
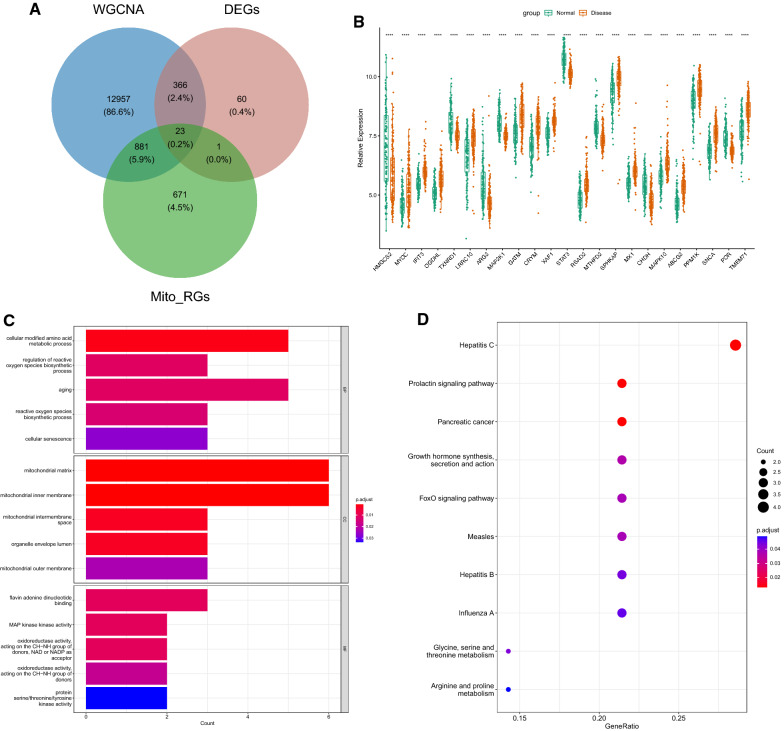
Identification and enrichment analysis of mitochondrial DEGs related to HF. **A** Venn diagram of DEGs, yellow module, and Mito-RGs; **B** Boxplot of 23 common genes in the Hf vs. the normal groups; **C** Go enrichment results (Top 5); **D** KEGG enrichment results (Top 10)

A total of 23 HF-Mito DEGs were obtained by crossover, and the Venn diagram is shown in Fig. 3A (Additional file 4: Table S4). A comparison analysis revealed that *MYOC*, *IFIT3*, *OGDHL*, *LRRC10*, *GATM*, *CRYM*, *XAF1*, *RSAD2*, *SPHKAP, MX1*, *MAPK10*, *ABCG2*, *PPM1K*, *SNCA*, and *TMEM71* were highly expressed in HF, whereas *HMGCS2*, *TXNRD1*, *ARG2*, *MAP2K1*, *STAT3*, *MTHFD2*, *CHDH*, and *POR* were poorly expressed (Fig. 3B). The GO and KEGG functional enrichment of the 23 HF-Mito DEGs yielded 46 BP (biological process), 8 cellular components (CCs), 6 molecular functions (MFs), and 10 KEGG signaling pathways. Figure 3C, D present the enrichment results of the top five GO and top 10 KEGG signaling pathways, respectively. The results demonstrated that these genes were enriched in cellular modified amino acid metabolism, ROS biosynthesis regulation, aging process, biosynthetic process, and other biological processes, and KEGG signaling pathways such as hepatitis C and prolactin signaling pathway.

### Identification of key genes and GSEA

**Fig. 4:**
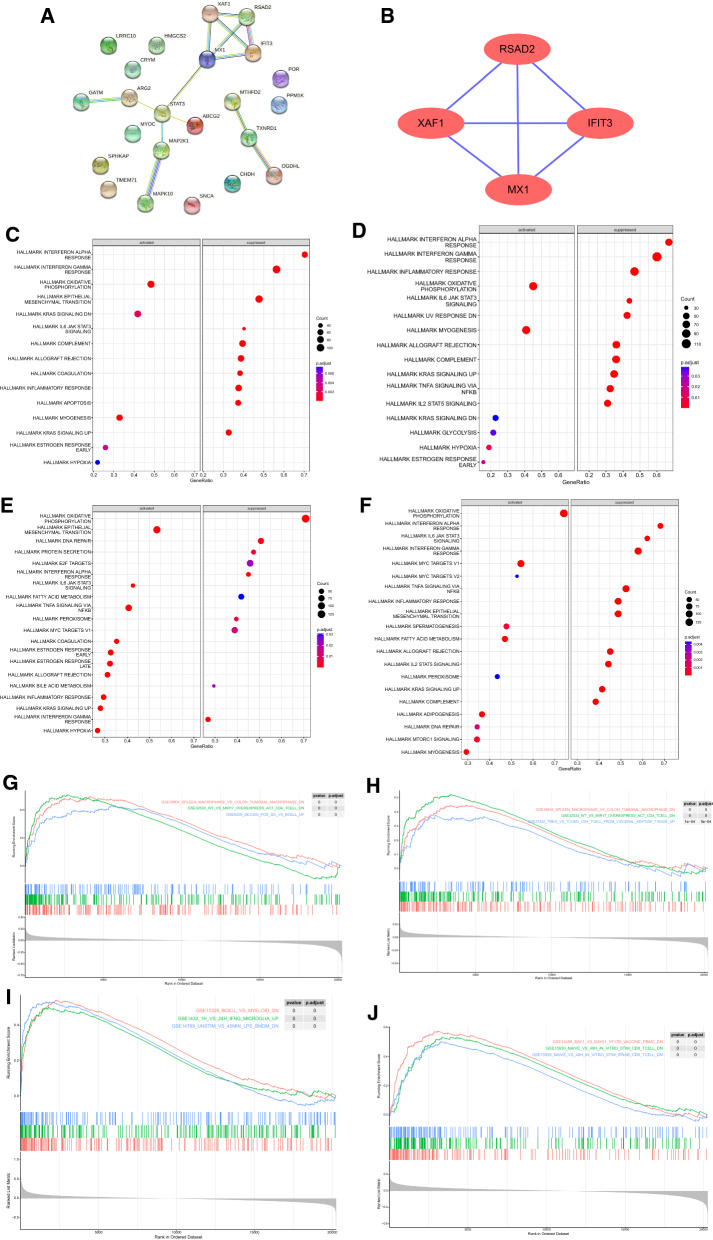
Identification of key genes and GSEA enrichment analysis. **A** PPI network display; **B** MCODE analysis results; **C–J** Hallmark and Immune enrichment results of key genes (IFIT3, MX1, RSAD2, and XAF1)

A PPI network was performed on the 23 HF-Mito DEGs using the STRING website. After removing discrete proteins (such as *HMGCS2*, *MYOC*, *LRRC10*, *CRYM*, *SPHKAP*, *CHDH*, *PPM1K*, *SNCA*, *PORh,* and *TMEM71*, because they did not interact with other proteins), 14 pairs were obtained. A gene network cluster was obtained using MCODE analysis as shown in Fig. 4A, including *IFIT3, XAF1*, *RSAD2*, and *MX1* key genes. The key genes were all upregulated genes (Fig. 4B). The median values of *IFIT3, XAF1, RSAD2*, and *MX1* genes were used to classify 177 HF patients into low- or high-expression group, and then GSEA analysis was performed for all genes. The top 10 hallmark and top three immune-related pathways for the four key genes are shown in Fig. 4C–J. *IFIT3* and *MX1* high-expression samples were enriched in myogenesis, KRAS signaling DN, oxidative phosphorylation, hypoxia, and estrogen response. Moreover, inflammatory response and *IL-6*, *JAK* expression were significantly enriched in *RSAD2* high-expression samples. IL-6/JAK/STAT3 signaling, oxidative phosphorylation, fatty acid metabolism, and other pathways were significantly enriched in* XAF1* high-expression samples.

### Immune cells scores analysis in HF and normal groups

**Fig. 5:**
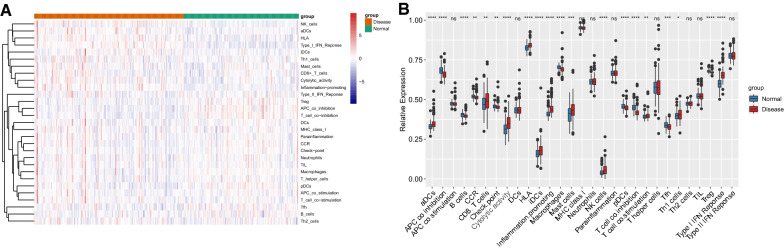
ssGSEA used for analyzing immunocyte infiltration between the normal and the HF groups. **A** Heat map; **B** Boxplot of 29 immune gene-sets content between HF vs. normal groups

**Fig. 6:**
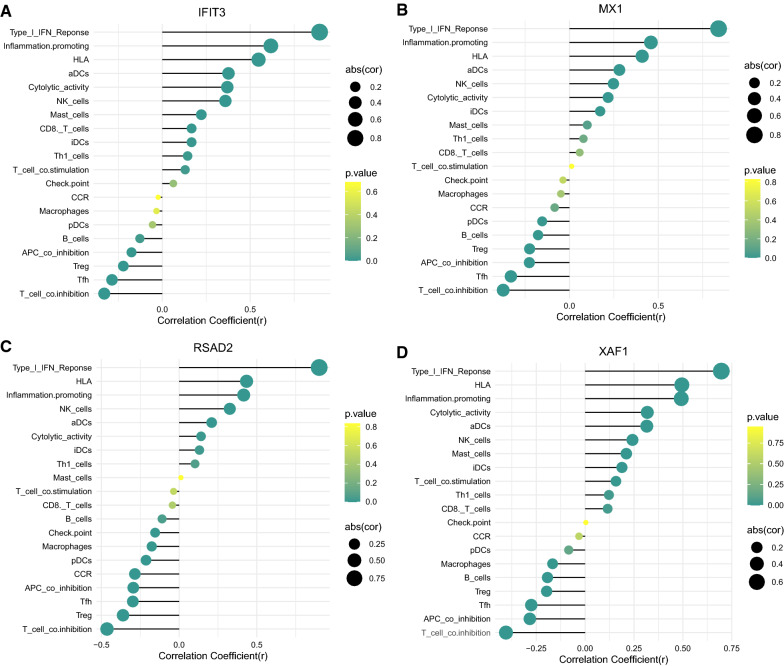
Association between critical genes with DEGs. **A–D** Correlation diagram for key genes and immune DEGs set (*IFIT3, XAF1, RSAD2,* and *MX1*)

Figure 5A, B show the heat map and box line plot of the 29 immune gene sets of all samples. aDCs, cytolytic activity, CD8+ T cells, HLA, iDCs, inflammation-promoting, NK cells, type I IFN response, mast cells, Th1 cells, and T cell co-stimulation showed high expression in the HF group, whereas B cells, APC co-inhibition, checkpoint, CCR, macrophages, pDCs, T cell co-inhibition, Tfh, and Treg were less expressed in the HF group. Moreover, the correlation plot of the key genes and the differential immune gene sets are shown in Fig. 6. *XAF1* positively correlated with inflammation-promoting, HLA, cytolytic activity, type I IFN response, NK cells, aDCs, iDCs, mast cells, CD8 T cells, Th1 cells, and T cell co-stimulation, whereas it negatively correlated with APC co-inhibition, T cell co-inhibition, macrophages, Tfh, B cells, and Treg. MX1 positively correlated with ADCs and inflammation-promoting, iDCs, HLA, Type I IFN response, cytolytic activity, and NK cells, but showed negative relation to APC co-inhibition, pDCs, Tfh, T cell co-inhibition, B cells, and Tregs. *RSAD2* positively correlated with aDCs, HLA, inflammation-promoting, type I IFN response, NK cells, iDCs, and cytolytic activity, and negatively correlated with T cell co-inhibition, Treg, APC co-inhibition, CCR, Tfh, pDCs, checkpoint, and macrophages.* IFIT3* positively correlated with HLA, ADCs, type I IFN response, inflammation-promoting, cytolytic activity, NK cells, mast cells, CD8+ T cells, iDCs, T cell co-stimulation, and Th1 cells, and negatively correlated with T cell co-inhibition, B cells, Tfh, Treg, and APC co-inhibition (Additional file 5: Table S5).

### Drug prediction analysis of the key genes

The current study used the CTD database to predict potential therapeutic agents for the key genes, and the results are shown in Additional file 6: Table S6. A total of 146 drugs were predicted by *IFIT3*, 142 by *MX1*, 144 by *RSAD2*, and 83 by *XAF1*.

### Critical genes-based competing endogenous RNA (ceRNA) network establishment

**Fig. 7:**
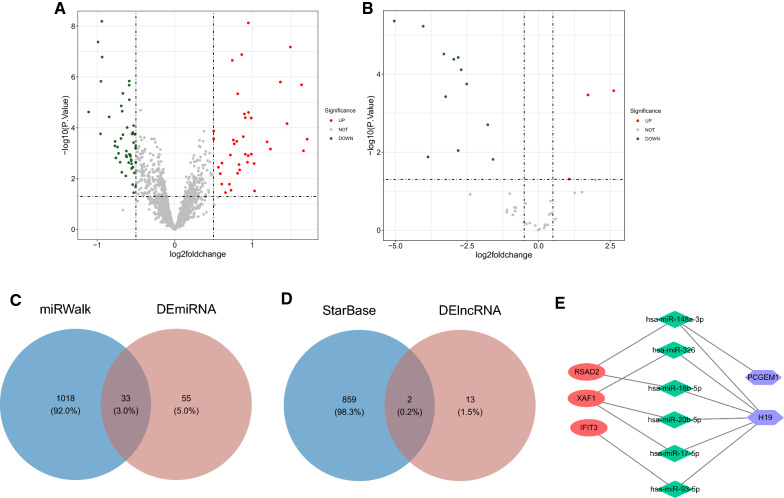
CeRNA regulatory network establishment based on critical genes. **A** Volcano plot showing differentially-expressed miRNA in the HF vs. the normal groups; **B** Volcano plot showing differentially-expressed lncRNA in the HF vs. the normal groups; **C** Venn diagram showing miRWalk and differentially-expressed miRNA; **D** Venn diagram of StarBase and differentially-expressed lncRNA; **E** ceRNA regulatory network (11Nodes-13Edges)

We observed 88 differential miRNAs in the GSE136547 dataset, including 39 upregulated miRNAs and 49 downregulated miRNAs (Additional file 7: Table S7 and Additional file 8: Table S8). Three lncRNAs were upregulated and 12 lncRNAs were downregulated in the GSE77399 dataset (Additional file 9: Table S9 and Additional file 10: Table S10). To understand the overall distribution of the differentially expressed lncRNAs and miRNAs, volcano plots are shown in Fig. 7A, B. miRWalk (http://mirwalk.umm.uni-heidelberg.de/) was utilized to predict the four key genes, resulting in 1051 miRNAs predicted to bind to their targets. A total of 33 common miRNAs were obtained by taking the intersection of these miRNAs with 88 differential miRNAs (Fig. 7C), followed by using starBase database to predict 12 miRNAs interacting with these 33 common miRNAs. The starBase database was used to predict 861 lncRNAs interacting with these 33 common miRNAs, and the predicted lncRNAs were intersected with 15 differential lncRNAs to obtain two common lncRNAs (Fig. 7D). Then, the final two lncRNAs (PCGEM1, H19) were used to extract six miRNAs with regulatory relationships (has-miR-148a-3p, has-miR-326, has-miR-17-5p, has-miR-18b-5p, has-miR-20b-5p, has-miR-93-5p) together with three key genes (*RSAD2*, *XAF1*, *IFIT3*). Finally, the network of three key genes, six miRNAs, and two lncRNAs was constructed (Fig. 7E).

### Validation of the key gene levels

**Fig. 8:**
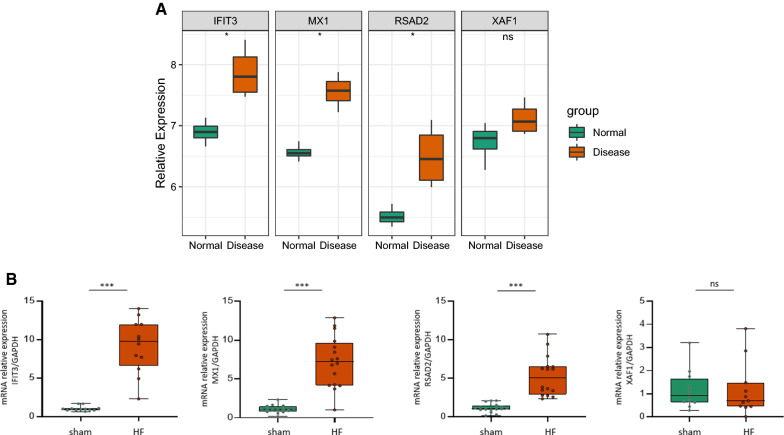
Verification of the expression level of key genes. **A** GSE76701 data set was used to verify the critical gene levels; **B** qRT-PCR verification for critical genes. Values represent means ± SEM, n = 12/group, ***P < 0.0001 vs. the control group

The four key genes, *IFIT3, XAF1, RSAD2*, and *MX1*, in the normal and HF groups, are shown in Fig. 8A. Wilcoxon test results indicated that *IFIT3, MX1*, and *RSAD2* were highly expressed in the HF group of the GSE76701 dataset, and the gene expression trends were completely consistent with those in the GSE57338 dataset. qRT-PCR verification was performed in the left ventricular tissue samples of the normal and HF groups to verify critical gene levels. Similar gene expression trend of XAF1 was observed (Fig. 8B).

## Discussion

1. The severity of HF is related to energy metabolism disorder. Mitochondrial function is particularly important in myocardial cells, which continually consume energy (HF is related to mitochondrial dysfunction). The treatment of HF requires expensive medical procedures. The development of accurate biomarkers and therapeutic targets can improve the level of diagnosis and treatment. The treatment scheme targeting the energy metabolism pathway can improve the degree of HF and heart function.
2. There are four discoveries in the current study. In brief, we obtained four key genes (*IFIT3, XAF1, RSAD2*, and *MX1*) through the bioinformatics analysis of 1576 Mito-RGs obtained from four datasets of GSE57338 (mRNA), GSE76701 (mRNA), GSE136547 (miRNA), and GSE77399 (mRNA), and literature in GEO database [6]. *IFIT3* (interference-induced protein with tetratricopeptide repeats 3) is a protein-coding gene. Typically, interferon gene serves as a possible biomarker to diagnose and treat ischemic cardiomyopathy [6]. Its effects on mitochondria-related factor VDAC2 and apoptosis were determined [13]. *XAF1* (XIAP-associated factor 1) activates the mitochondrial apoptosis pathway and is used as a proapoptotic factor in treating cerebral ischemia–reperfusion injury, cancer prognosis, and atherosclerosis of the aorta [14]. *RSAD1* (radical S-adenosylmethionine domain-containing 1), also known as viperin, belongs to the S-adenosine-L-methionine (SAM) enzyme superfamily. *RSAD1* can widely activate E3 ligase and increase proteasome-mediated protein degradation upon virus infection [15]. The genomic interval plays a vital role in heart health [16]. These gene deletions cause congenital heart diseases. The mechanism of RSAD1 on HF has not been studied deeply; however, its effect on the heart and its wide distribution in mitochondria can provide new ideas for studying HF. *MX1* (MX dynamin-like GTPase) is a gene that encodes a guanosine triphosphate (GTP) metabolic protein involved in cell antiviral response. In this study, we used Wilcoxon paired test method to verify the absence of distinct differences in its level within the GSE76701 dataset. Interestingly, the change in *MX1* gene expression was also screened [17]; yet, *MX1* expression was not defined as related to cardiac function.
3. Furthermore, our enrichment analysis (GO and KEGG) revealed the significant enrichment of such genes into biological processes such as cellular modified amino acid metabolism, ROS biosynthesis regulation, and aging, and signal pathways, such as hepatitis C and prolactin signaling pathway. In terms of the mechanism, the characteristics of HF include aberrant energy metabolism, enhanced ROS generation, and a defect in excitation–contraction coupling. As the pathological condition with high dynamics, HF shows changes in cardiac function and metabolism during the disease progression. Research on HF in mitochondria includes MPTP opening, mitochondrial autophagy, and mitochondrial unfolded protein response (caused by an oxidative metabolic disorder, calcium overload, mitochondrial fusion, and fission). The metabolic redistribution of cardiomyocytes is also a research direction for HF treatment, such as the decrease of mitochondrial pyruvate oxidation, the increase of lactate output, and the protective effect of plasma amino acid metabolic spectrum on the failing heart. The prevalence of HF in older adults is increasing significantly. The relationship of HF and aging is related to oxidative stress. Mitochondria are the primary ROS source in cells and are considered the central controller of the aging process. The cardiovascular aging process is mainly regulated by risk factors, preexisting diseases, and age-related factors. Therefore, targeted treatment may delay the aging process or improve its complications.
4. We analyzed 20 immune gene sets with significant differences through single-sample GSEA (ssGSEA). Proinflammatory cytokines (IL-6, TNFα, NF-κB, etc.) have been associated with the course of HF. They can also further aggravate the disease process of HF through apoptosis. Macrophages and T lymphocytes also play an important role in HF, which further explains HF and the role of immune genes.
5. We used miRWalk and StarBase databases to predict six miRNAs and two lncRNAs for the network construction. Yet, no HF-relevant report was available for these molecules, which is expected to be further explored in subsequent experiments.
6. In this study, we screened four key genes, including *IFIT3*, *XAF1*, *RSAD2*, and *MX1*. Through examining the relation of the key genes with differential immune cells, we predicted therapeutic drugs of the four genes and constructed the key genes–based ceRNA network. In subsequent research, we can further analyze the four key genes’ specific molecular mechanisms within HF.

## Supplementary Information

**Additional file 1: Table S1.** Volcano plot upregulated gene dataset.

**Additional file 2: Table S2.** Volcano plot downregulated gene dataset.

**Additional file 3: Table S3.** Module data set.

**Additional file 4: Table S4.** Venn diagram dataset.

**Additional file 5: Table S5.** Key genes and immune correlation dataset.

**Additional file 6: Table S6.** CTD database sources key genes.

**Additional file 7: Table S7.** Upregulated miRNA dataset.

**Additional file 8: Table S8.** Downregulated miRNA dataset.

**Additional file 9: Table S9.** Lncrna upregulated gene dataset.

**Additional file 10: Table S10.** Lncrna downregulated gene dataset.

## Abbreviations

ATP: Adenosine triphosphate
BP: Biological process
CCs: Cellular components
ceRNA: Competing endogenous RNA
CTD: Comparative toxicogenomics database
DEGs: Differentially expressed genes
ECG: Electrocardiogram
FC: Fold change
GEO: Gene expression omnibus
GO: Gene ontology
GSEA: Gene set enrichment analysis
GTP: Guanosine triphosphate
HF: Heart failure
HFGs: HF-related genes
KEGG: Kyoto Encyclopedia of Genes and Genomes
MCODE: Molecular complex detection
MFs: Molecular functions
miRNA: MicroRNA
Mito-RGs: Mitochondria-related genes
MMP: Mitochondrial membrane potential
MPTP: Mitochondrial permeability transition pore
MSigDB: Molecular signatures database
PCA: Principal components analysis
PPI: Protein–protein interaction
ROS: Reactive oxygen species
SAM: S-adenosine-L-methionine
ssGSEA: Single-sample GSEA
TCA: Tricarboxylic acid
WGCNA: Weighted gene co-expression network analysis
WT: Wild-type
